# Cigarette smoke preferentially induces full length ACE2 expression in differentiated primary human airway cultures but does not alter the efficiency of cellular SARS-CoV-2 infection

**DOI:** 10.1016/j.heliyon.2023.e14383

**Published:** 2023-03-11

**Authors:** Linsey M. Porter, Wenrui Guo, Thomas WM. Crozier, Edward JD. Greenwood, Brian Ortmann, Daniel Kottmann, James A. Nathan, Ravindra Mahadeva, Paul J. Lehner, Frank McCaughan

**Affiliations:** a Department of Medicine, University of Cambridge, Addenbrookes Hospital, Cambridge, CB2 OQQ, UK; b Cambridge Institute of Therapeutic Immunology & Infectious Disease, Department of Medicine, University of Cambridge, Puddicombe Way, Cambridge, CB2 0AW, UK; c Cambridge University Hospitals NHS Foundation Trust, University of Cambridge, Addenbrookes Hospital, Cambridge, CB2 OQQ, UK

**Keywords:** SARS-CoV-2, Primary human bronchial epithelial cells, ACE2, Air-liquid interface, Cigarette smoke extract

## Abstract

Cigarette smoking has many serious negative health consequences. The relationship between smoking and SARS-CoV-2 infection is controversial, specifically whether smokers are at increased risk of infection. We investigated the impact of cigarette smoke on ACE2 isoform expression and SARS-CoV-2 infection in differentiated primary human bronchial epithelial cells at the air-liquid-interface (ALI). We assessed the expression of ACE2 in response to CSE and therapeutics reported to modulate ACE2. We exposed ALI cultures to cigarette smoke extract (CSE) and then infected them with SARS-CoV-2. We measured cellular infection using flow cytometry and whole-transwell immunofluorescence. We found that CSE increased expression of full-length ACE2 (flACE2) but did not alter the expression of a Type I-interferon sensitive truncated isoform (dACE2) that lacks the capacity to bind SARS-CoV-2. CSE did not have a significant impact on key mediators of the innate immune response. Importantly, we show that, despite the increase in flACE2, CSE did not alter airway cell infection after CSE exposure. We found that nicotine does not significantly alter flACE2 expression but that NRF2 agonists do lead to an increase in flACE2 expression. This increase was not associated with an increase in SARS-CoV-2 infection. Our results are consistent with the epidemiological data suggesting that current smokers do not have an excess of SARS-CoV-2 infection. but that those with chronic respiratory or cardiovascular disease are more vulnerable to severe COVID-19. They suggest that, in differentiated conducting airway cells, flACE2 expression levels may not limit airway SARS-CoV-2 infection.

## 1 Introduction

SARS-CoV-2 is the causative agent of coronavirus disease 2019 (COVID-19). The SARS-CoV-2 envelope spike (S) protein is essential for virus attachment and cell entry via the main cellular receptor - angiotensin converting enzyme 2 (ACE2) [1]. Entry is further dependent on S-protein priming by TMPRSS2 facilitating fusion of viral and cellular membranes [2] or via the endosomal pathway.

ACE2 is a membrane-associated aminopeptidase expressed in a range of tissues [3]. In the renin-angiotensin-aldosterone system (RAAS), it converts the vasoconstrictive hormone angiotensin-II to the vasodilator Ang 1–7 but has other physiological roles [4].

The physiological role for ACE2 at homeostasis in airway epithelial cells is unknown. In preclinical murine models ACE2 was confirmed as the receptor for SARS-CoV-1 and conferred protection against SARS-CoV-1 associated lung injury [5]. However, the mechanism by which ACE2 mediates protection is unclear and may relate to its role in the pulmonary vascular endothelium rather than the airway epithelium [6]. In transcriptomic studies of the human respiratory tract and lung, there is a proximal-distal ACE2 mRNA expression gradient; expression is highest in the nasal epithelium and lower distally in the alveolar epithelium, mirroring the permissiveness to SARS-CoV-2 infection [[7], [8], [9]]. The distribution of ACE2 protein expression is less well characterised due to the paucity of validated reagents but is consistent with the proximal-distal graded expression pattern [3,10]. Notably, ACE2 expression in the lung is not altered by ACE inhibitors or angiotensin receptor blockers [11].

Recently, an N-terminally truncated (dACE2) isoform that is sensitive to interferon stimulation or viral infection has been detected [[12], [13], [14]]. Importantly, dACE2 does not express the SARS-CoV-2 spike-protein binding domain and its relevance in SARS-CoV-2 infection and normal physiology remains unclear [12,14].

The impact of ACE2 expression on COVID-19 incidence and severity is also unclear [15]. Much of the available information on ACE2 expression is from scRNA databases and has not reported the relative expression of the two isoforms (for 3-prime scRNA-Seq they will be indistinguishable). Given the differential isoform binding to SARS-CoV-2, this is likely to be an important issue. More recently a study has examined protein expression of both isoforms in multiple tissues and reported relatively enriched expression of dACE2 in lung and liver tissue [16].

Smoking has been associated with increased ACE2 expression in multiple studies including in patients with COPD [[17], [18], [19], [20], [21]] and in airway cells from occasional or “social” smokers exposed to 3 cigarettes over a 24hr period [22]. Importantly, studies to date have not linked RNASeq data with protein and isoform expression.

The epidemiological data regarding the association of smoking with COVID-19 is conflicting and controversial [[23], [24], [25], [26], [27], [28]]. However, a ‘living’ meta-analysis suggests that current smoking is not associated with an increased risk of SARS-CoV-2 infection [24]. Large surveys have suggested that chronic respiratory disease including COPD may be associated with an increased risk of severe COVID-19 [29,30]. This is consistent with the observation that former smokers were at an increased risk of severe COVID-19 [24,31].

Differentiated human airway cells grown at the air-liquid-interface (ALI) recapitulate the pseudostratified airway of the *in vivo* airway and are the best laboratory system to model the impact of smoking on the early stages of SARS-CoV-2 infection [14,32]. Purkayastha and colleagues recently reported brief exposure of primary airway cultures (3 min/day for four days) to “headspace” cigarette smoke (CS), that is CS in a closed environment, to investigate the impact of CS on ACE2 expression and SARS-CoV-2 infection [32]. In contrast to the published molecular epidemiological data discussed above [17,18], there was no increase in ACE2 expression in response to CS. However, they reported that CS exposure led to an increase in viral infection at 48 h.

We now report our results from experiments in which we exposed differentiated multicellular primary human bronchial epithelial cells (HBECs) at ALI to cigarette smoke extract (CSE). We show that HBECs upregulate ACE2 expression in response to CSE – consistent with the molecular epidemiological evidence [17,18]. However, this did not lead to an increase in infection by SARS-CoV-2 despite preferential upregulation of full-length ACE2 receptor (flACE2) rather than the N-terminal truncated isoform in response to CSE exposure. This suggests that in differentiated normal human bronchial epithelial cells, the level of expression of flACE2 does not limit viral infectivity. We go on to show that dACE2 is a Type I interferon-sensitive gene in primary HBECs, and define the impact of CSE, nicotine and NRF2 agonists on ACE2 isoform expression.

This study directly addresses one of the controversies in the link between smoking and SARS-CoV-2. Our results are consistent with epidemiological evidence suggesting that current smoking is not associated with a higher incidence of SARS-CoV-2 infection.

## 2 Materials and methods

### 2.1 Primary human bronchial epithelial cell (HBEC) culture and other cell line culture

Primary human bronchial epithelial cells (HBECs) derived from a non-smoking donor (Cat# CC-2540, male; Lonza; Donor 1) or derived directly from a patient at Cambridge University Hospitals NHS Trust (Research Ethics Committee Reference 19/SW/0152; Donor 2) were expanded using PneumaCult™-Ex Plus Medium (Cat# 05040; Stemcell) supplemented with Penicillin (100 I.U./ml)-Streptomycin (100 μg/ml). All experiments were performed using cells at passage 3. All experiments in used cells expanded from Donor 1 except when stated otherwise.

Details of the cell lines used as positive or negative controls are included in the supplementary material.

### 2.2 Air-liquid interface (ALI) culture

ALI cultures were established from primary bronchial epithelial cells as described previously [33]. Briefly, 1 × 10^5^ of expanded primary HBECs at passage 3 in 200 μl of supplemented PneumaCult™-Ex Plus Media were seeded in the apical chamber of a 24-well Transwell® insert with 0.4 μM pore (Cat# 353095, Falcon) pre-coated with Rat tail Type I collagen (Cat# 354236, Corning) with 500 μl of PneumaCult™-Ex Plus Media in the basolateral chamber. The following day, both apical and basolateral chambers underwent a media change (200 μl and 500 μl, respectively). After two days of submerged culture, media from the apical chamber was removed to establish the air-liquid interface (ALI day 0) whilst media in the basolateral chamber was replaced with 500 μl HBEC ALI differentiation medium (PneumaCult™-ALI Medium, Cat# 05021; Stemcell). Basolateral media was changed every 2–3 days and apical surface washed with warm PBS twice a week to remove any build-up of mucous and secretions. Cultures were allowed to differentiate for at least 28 days before being used for any experiments.

### 2.3 Cigarette smoke extract (CSE) generation and treatment

Cigarette smoke extract (CSE) was prepared, filter sterilised using 0.20 μm filter and used within 30 min of generation. CSE was generated by smoking two Kentucky reference cigarettes and bubbling the generated smoke through 25 ml ALI media at a rate of 100 ml/min. Each cigarette took roughly 6 min to burn. This solution is regarded as “100% CSE” and was diluted with ALI media to generate a 10% working solution. Cells were treated with 10% CSE for 48 h before being treated with SARS-CoV-2, harvested or fixed for further analysis.

### 2.4 SARS-CoV-2 infection

A standard infection protocol using clinical isolates was used as before [33] The SARS-CoV-2 viruses used in this study were named “SARS-CoV-2/human/Liverpool/REMRQ0001/2020” (Lineage B variant (B.29) from a patient in February 2020 that lacked the D614G mutation in S protein) [[34], [35], [36]] and “SARS-CoV-2 England/ATACCC 174/2020” (Lineage B.1.1.7) [35,37]. Viral stocks were obtained from collaborators as detailed in the acknowledgments. Stocks were sequenced before use and the consensus matched the expected sequence exactly. Viral titre was determined by 50% tissue culture infectious dose (TCID50) in Huh7-ACE2 cells.

ALI cultures were exposed to virus for 2–3 h and assays (flow cytometry, immunofluorescence) performed at 72 h. The 72 h timepoint was chosen based on data from our recently published work in which we performed an infection timecourse (10.12688/wellcomeopenres.17946.1) which was very similar to published data [38].

For infection, the indicated dose of virus was diluted in PBS to a final volume of 50 μL and added to the apical chamber of the transwell of differentiated HBEC-ALI cultures for 2–3 h prior to removal. At 72 h post-infection HBEC-ALI apical surfaces were washed once with PBS, dissociated with TrypLE, and fixed in 4% formaldehyde for 15 min. Fixed cells were washed and incubated for 15 min at room temperature in Perm/Wash buffer (BD #554723). Permeabilised cells were pelleted, stained for 15 min at room temperature in 100 μL of sheep anti-SARS-CoV-2 nucleocapsid antibody (MRC-PPU, DA114) at a concentration of 0.7 μg/ml, washed and incubated in 100 μL AF488 donkey anti-sheep (Jackson ImmunoResearch #713-545-147) at a concentration of 2 μg/ml for 15 min at room temperature. Stained cells were pelleted and fluorescence staining analyzed on a BD Fortessa flow cytometer.

### 2.5 Immunofluorescence

A standard ALI immunofluorescence protocol was used as previously described [33,39]. Whole transwell immunofluorescence quantitation was also performed essentially as previously described [33]. The details of the protocol and whole transwell immunofluorescence are in the supplementary material.

### 2.6 QPCR

A standard protocol was used, essentially as previously described. In brief, RNA was extracted using RNeasy Mini Kit (Qiagen) according to manufacturer's instructions. cDNA synthesis was performed using a High-Capacity cDNA Reverse Transcription Kit (ThermoFisher). qRT-PCR was performed using Fast SYBR® Green Mix (ThermoFisher) alongside the following primers used for genes of interest: ACE2 Forward (5′-3′): CGAAGCCGAAGACCTGTTCTA, Reverse (5′-3′): GGGCAAGTGTGGACTGTTCC; dACE2 Forward (5′-3′): GGAAGCAGGCTGGGACAAA, Reverse (5′-3′): AGCTGTCAGGAAGTCGTCCATT; TBP (House keeper) Forward (5′-3′): AGTGAAGAACAGTCCAGACTG, Reverse (5′-3′): CCAGGAAATAACTCTGGCTCAT; TMPRSS2 Forward (5′-3′): CTGCTGGATTTCCGGGTG, Reverse (5′-3′) TTCTGAGGTCTTCCCTTTCTCCT; FOXJ1 Forward (5′-3′): TGGATCACGGACAACTTCTGCTA, Reverse (5′-3′) CACTTGTTCCAGAGACAGGTTGTGG; MUC5B Forward (5′-3′): CCTGAAGTCTTCCCCAGCAG, Reverse (5′-3′) GCATAGAATTGGCAGCCAGC. The expression level of a gene of interest was quantified using SYBR Green I Dye (Life Technologies) on a QuantStudio 7 Flex Real-Time PCR System (Applied Biosystems). The data was analyzed by Applied Biosystems Design and Analysis Software version 2.5 using the 2^−ΔΔCt^ method.

### 2.7 Western blotting

Standard western plotting protocols were followed. Recombinant Anti-ACE2 antibodies (Cat# ab108209; Abcam: N-terminal) and (Cat# ab15348; Abcam: C-terminal), alpha-tubulin (Cat# sc-32293; Santa-Cruz) and beta-actin (Cat# sc-69879; Santa-Cruz) were used for ACE2/dACE2, alpha-tubulin detection and beta-actin, respectively.

### 2.8 Apoptosis detection

ALI cultures exposed to CSE or control media were washed three times with PBS and detached from the transwell membrane with accutase. Apoptotic cells was detected by concurrent staining with annexin V–APC and PI (Cat# 88-8007-72, eBioscience) and their far-red and red fluorescence was measured by flow cytometry (Fortessa LSR, BD).

### 2.9 Quantification and statistical analysis

Statistical analyses of mRNA expression assays and infection quantification data were performed using Prism 8 software (GraphPad Software). P values were calculated using a two-tailed, Mann Whitney *U* test unless stated otherwise. P values were noted as follows: ns, not significant; *, P < 0.05; **, P < 0.01; ***, P < 0.001. Error bars represent the mean ± standard error of the mean unless stated otherwise.

## 3 Results

### 3.1 ACE2 is expressed on differentiated ciliated cells at homeostasis

**Fig. 1:**
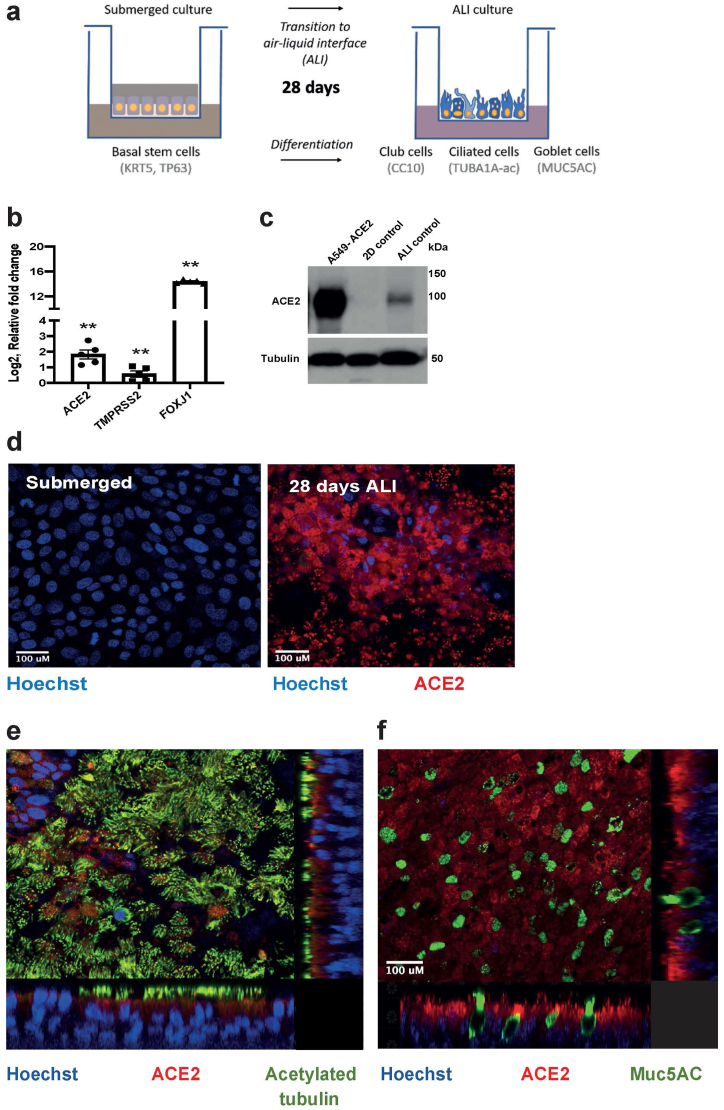
ACE2 expression increases upon differentiation in HBECs cultured at the air-liquid interface (ALI). **A.** Schematic representation of ALI experimental set-up using HBECs. Cell type specific markers are shown in parentheses. **B.** HBEC *TMPRSS2* and *ACE2* expression (RNA) both increase when cultured for 28 days at the ALI compared to submerged, non-differentiated cell culture. Expression of the transcription factor required for ciliation, *FOXJ1* is also significantly upregulated. RT-qPCR data presented as log2 relative fold-change in expression compared to submerged HBECs from n = 5 independent experiments (Mann Whitney, **, P < 0.01). Error bars represent mean and the standard error of the mean. **C.** ACE2 protein is also increased during differentiation. A549 cells overexpressing ACE2 are used as a positive control. Representative Western blot from 3 independent experiments. ACE2 antibody Ab228349 was used. **D.** ACE2 expression (red fluorescence, antibody Ab228349) is upregulated on differentiation. **E**) ACE2 (red, Antibody used 21115AP) is expressed apically on the epithelial cell surface, predominantly colocalising with ciliated cells (acetylated tubulin, green fluorescence). **F) ACE2** (red, antibody 21115AP) does not colocalise with goblet cells (MUC5AC, green fluorescence). Scale bars on fluorescent images = 100 μm. All experiments in Fig. 1 used cells expanded from Donor 1.

Previous studies have shown ACE2 expression increases following differentiation at ALI, but could be reversed if cultures were resubmerged [40]. We grew HBECs from Donor 1 at ALI for a minimum of 4 weeks to produce a well-differentiated, pseudostratified mucociliary epithelium (Fig. 1a). ACE2 mRNA expression was increased on differentiation, as was TMPRSS2, the main cellular protease implicated in SARS-CoV-2 spike protein cleavage and FOXJ1, a key transcription factor regulating airway ciliation. Full length ACE2 protein was not detectable in submerged HBEC cultures but was readily detectable on differentiation (Fig. 1b–d) emphasising the importance of using differentiated HBECs to model airway infection. Confocal imaging demonstrated apical ACE2 expression colocalising with markers of ciliated but not goblet cells (Fig. 1 e,f).

### 3.2 Cigarette smoke extract increases ACE2 expression in differentiated HBECs

**Fig. 2:**
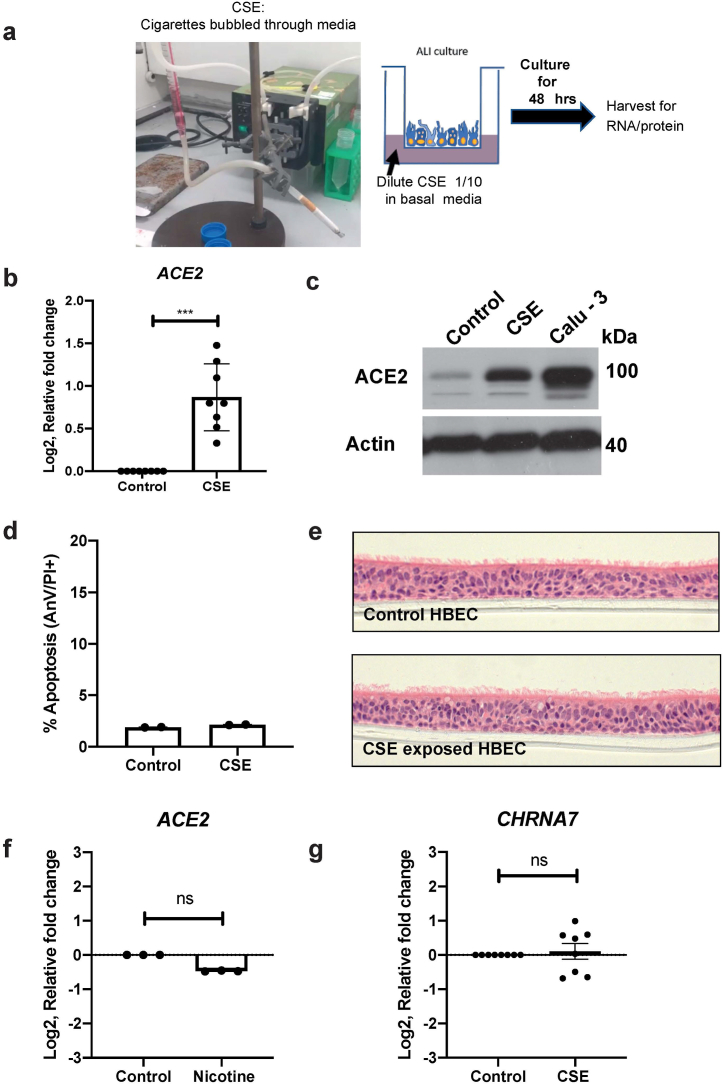
Exposure to cigarette smoke extract (CSE) increases ACE2 expression at both the RNA and protein level **A.** Schematic showing ALI culture experimental set-up with CSE. **B.** CSE exposure (10%) for 48 h increases *ACE2* expression in differentiated HBECs relative to untreated controls. RT-qPCR data presented as log2 relative fold-change in expression from n = 8 independent experiments (Mann-Whitney, ***, P < 0.001). **C.** CSE exposure (10%) for 48 h also increases ACE2 protein expression relative to untreated control. Calu-3 are used as the positive control as they robustly express ACE2. Representative Western blot from 3 independent experiments. ACE2 antibody – ab15348. **D.** CSE exposure does not induce apoptosis in differentiated HBECs at ALI relative to control samples as analyzed by flow cytometric analysis using AnnexinV (AnV) and Propidium iodide (PI) staining see also Supplementary Fig. 4. Data represents 2 independent experiments using cells from Donor 1. **E.** H&E staining of sectioned differentiated ALI HBEC cultures from Donor 1 with and without CSE exposure (×20 magnification). **F**. Treatment of differentiated HBECs at ALI with 1 μM Nicotine for 48 h does not induce *ACE2* expression. **G**. CSE exposure does not significantly alter *CHRNA7* expression **G**. RT-PCR data for **F** and **G** is presented as log2 relative fold-change in expression from n = 3–8 independent experiments (Mann-Whitney, ns).

We tested 10–20% cigarette smoke extract (CSE) and performed a timecourse to ensure the optimal time-point for infection after CSE exposure (Supplementary Fig. 1). We then exposed differentiated primary HBEC ALI cultures from Donor 1–10% cigarette smoke extract (CSE) for 48 h before harvesting cells (Fig. 2a). CSE exposure induced a significant increase in ACE2 (mRNA) and marked induction at the protein level relative to controls (Fig. 2b and c). We evaluated ACE2 immunofluorescence after CSE exposure which was consistent with increased apical ACE2 expression relative to control wells (Supplementary Fig. 2). Increased ACE2 levels were also detected from differentiated ALI cultures derived from Donor 2, a former smoker (61 pack-years) with COPD (Supplementary Fig. 3). Of note, there was no evidence of cytotoxicity in response to CSE, as exposure did not cause an increase in apoptosis or necrosis as shown by flow cytometric analysis (Fig. 2d; Supplementary Fig. 4) or an obvious cytopathic effect on histology (Fig. 2e). Importantly, given prior data on the impact of nicotine on ACE2 expression in submerged undifferentiated bronchial epithelial cells [41], we found that nicotine did not significantly alter the expression (mRNA) of either ACE2 or lead to a consistent change in ACE2 protein expression (Supplementary Fig. 5). CSE did not significantly affect expression of the key nicotinic acetylcholine receptor expressed on airway cells – CHRNA7 (Fig. 2f and g).

### 3.3 HBECs exposed to CSE are not more susceptible to infection by SARS-CoV-2

**Fig. 3:**
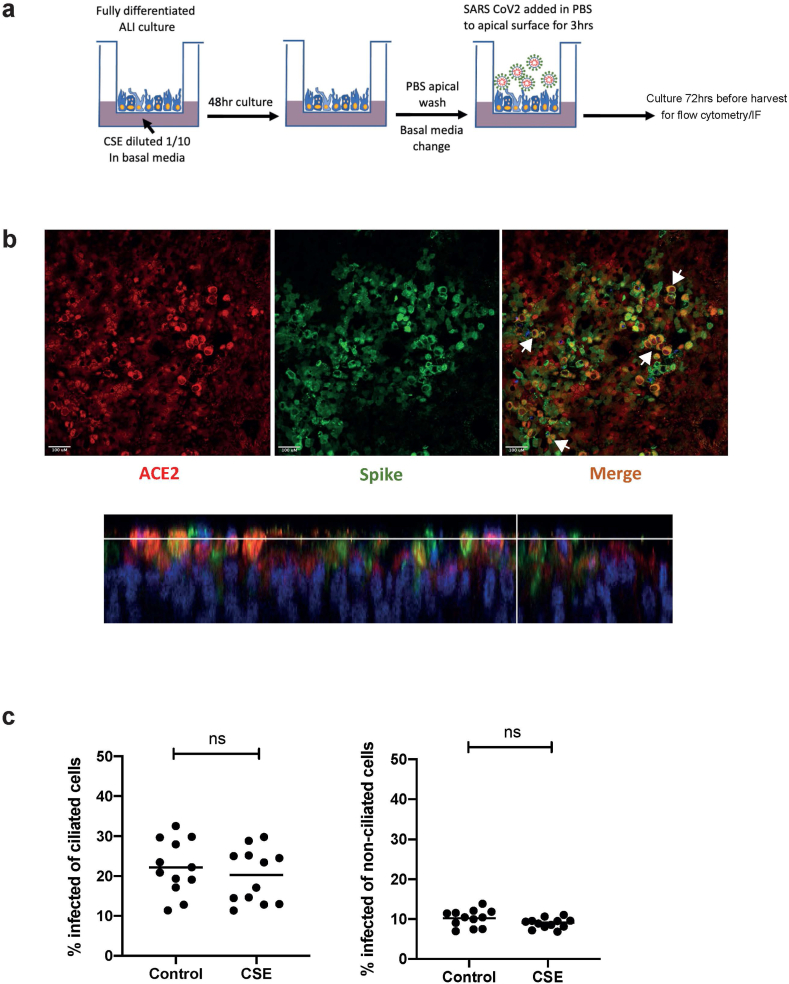
HBECs exposed to CSE are not more susceptible to infection by SARS CoV2 **A.** Schematic representation of SARS-CoV-2 infection of CSE exposed HBEC ALI cultures. **B.** SARS-CoV-2 infection was detected using an antibody specific to the viral spike protein (S2 domain). Representative immunofluorescent images showing 72 h post SARS-CoV-2 infection; expression of viral spike protein (green) primarily co-localised with ACE2 (red) expressing cells. White arrowheads indicate co-localisation of markers in merged imaged. **C.** Flow cytometry quantification of ciliated and non-ciliated cells infected with 8 × 10^3^ TCID50 of B.29 lineage SARS-CoV-2 following control/CSE exposure (n = 12); each dot represents an individual transwell from 2 independent experiments, bars represent mean values (Mann Whitney; ns, non-significant).

To determine whether CSE exposure would render the cells more susceptible to SARS-CoV-2 infection, differentiated ALI cultures (Donor1) were pre-treated with CSE for 48 h, then inoculated with SARS-CoV2 for 3 h and harvested after 72 h for flow cytometric quantitation of infection or immunofluorescence (IF) (Fig. 3a).

ACE2 colocalised with spike protein in infected wells (Fig. 3b) consistent with ciliated cells being more susceptible to coronavirus infection and with prior reports [42,43]. On some specimens infected cells appeared to be extruded from the epithelial surface – as previously reported for SARS-CoV-1 [42].

Importantly, despite the increased ACE2 levels following CSE exposure, there was no significant difference in the total infected fraction or infected ciliated cell fraction between control or CSE exposed cells (Fig. 3c). Therefore, in this model CSE exposure did not increase SARS-CoV-2 infection.

### 3.4 Regulation of ACE2 isoform expression – impact of cigarette smoke

The relationship between cigarette smoke exposure and viral infections has previously been investigated using primary cells – for example cigarette smoke was associated with increased rhinovirus infection in submerged cultures [44] or influenza A infection in differentiated ALI cultures [45]. A truncated isoform of ACE2 (dACE2) was recently implicated as an interferon-sensitive gene (ISG), but cannot act as a receptor for SARS-CoV-2 based on the ACE2-Spike receptor binding domain interaction [14,46].

**Fig. 4:**
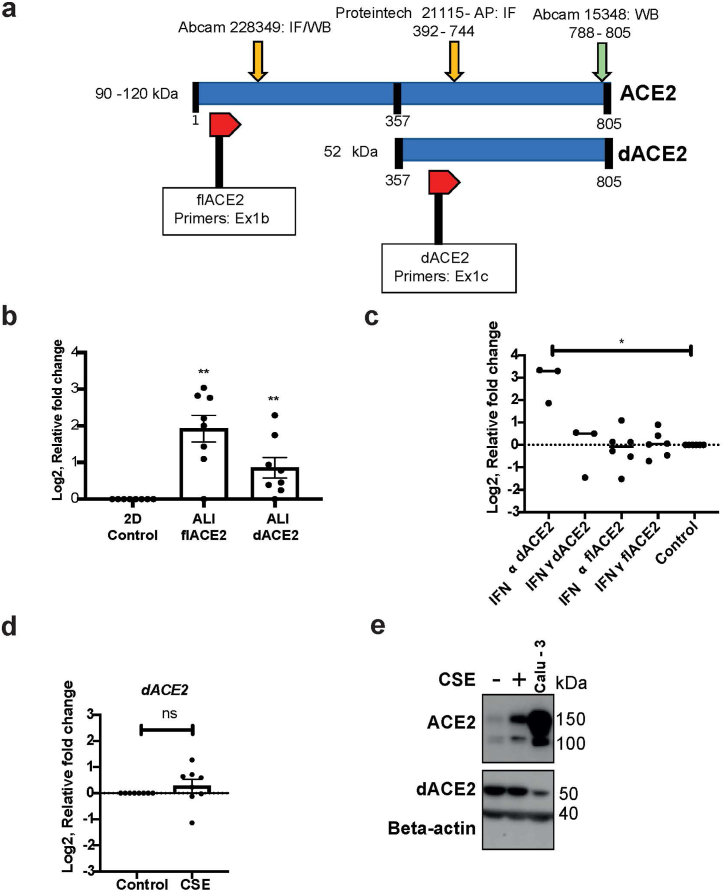
A short isoform of ACE2 is upregulated during HBEC differentiation and interferon-alpha stimulation but not CSE exposure at ALI. **A.** Schematic of full-length ACE2 (flACE2) and the truncated isoform (dACE2) detailing position of antibody binding epitopes for immunofluorescence and Western blot analysis. The location of primers used to distinguish ACE2 and dACE2 are also shown. Primer names are those originally described Onabajo et al., 2020 and relates to exon of both proteins. In this case Ex1b and Ex1c refers to primers designed for the flACE2 (Exon 1 b) and dACE2 (Exon 1c) in Onabajo et al., 2020 **B.** HBECs differentiated at ALI upregulate a short isoform of ACE2 (dACE2) as well as the full length ACE2. RT-PCR data shows log2 relative fold-change in expression from n = 7 independent experiments (Mann-Whitney **, P < 0.01). **C.** d*ACE2* is specifically sensitive to interferon-alpha stimulation (24 h) but not interferon-gamma at 24 h. Full-length ACE2 shows no modulation with interferon treatment at 24 h. RT-PCR data shows log2 relative fold-change in expression from n = 3–6 independent experiments (Mann-Whitney, *p < 0.05). **D**. 48 h exposure of CSE does not promote an increase in dACE2 mRNA. RT-PCR data shows log2 relative fold-change in expression from n = 7 independent experiments (Mann-Whitney, ns). **E**. Western blot is representative of 5 independent experiments and shows the impact of 48 h exposure of CSE on flACE2/dACE2 expression. Also see Supplementary Fig. 7. ACE2 antibody used ab15348.

We therefore explored the impact of cigarette smoke exposure on ACE2 isoform expression using recently described tools (Fig. 4a) [12]. We first assessed the relative expression of flACE2 and dACE2 in submerged primary HBECs compared to differentiated HBECs at ALI. dACE2 was upregulated on differentiation but modestly compared to the full-length receptor (Fig. 4b).

We next tested isoform-specific expression of ACE2 exposed HBECs to Type I and Type II interferons and CSE. IFN-α (Type I) but not IFN-γ (Type II) led to a transcriptional upregulation of dACE2 (mRNA). Neither interferon significantly altered the expression of the full-length transcript (Fig. 4c). This extends the recently published data from immortalised airway cells [14] and confirms that the N-terminus truncated transcript (dACE2) is an interferon-sensitive isoform. Further, CSE did not significantly alter the expression of a panel of interferon-sensitive genes (Suppl Fig. 6). Therefore, differentiated airway epithelial cells have the capacity to respond to Type I interferon and CSE does not mimic that response.

CSE did not significantly alter the expression of dACE2 mRNA (Fig. 4d). In agreement with the transcriptional data, both N & C-terminus ACE2 antibodies (Fig. 4a) showed that CSE consistently upregulated flACE2 protein but had no impact on an ACE2 band migrating at 52kd - the predicted molecular weight of dACE2 (Fig. 4e and Suppl Fig. 7) [14]. We conclude from these experiments that cigarette smoke does not activate interferon signalling or ISGs in normal human bronchial epithelial cells and preferentially upregulates flACE2 rather than dACE2.

### 3.5 Antioxidants upregulate ACE2 in differentiated airway epithelial cells

**Fig. 5:**
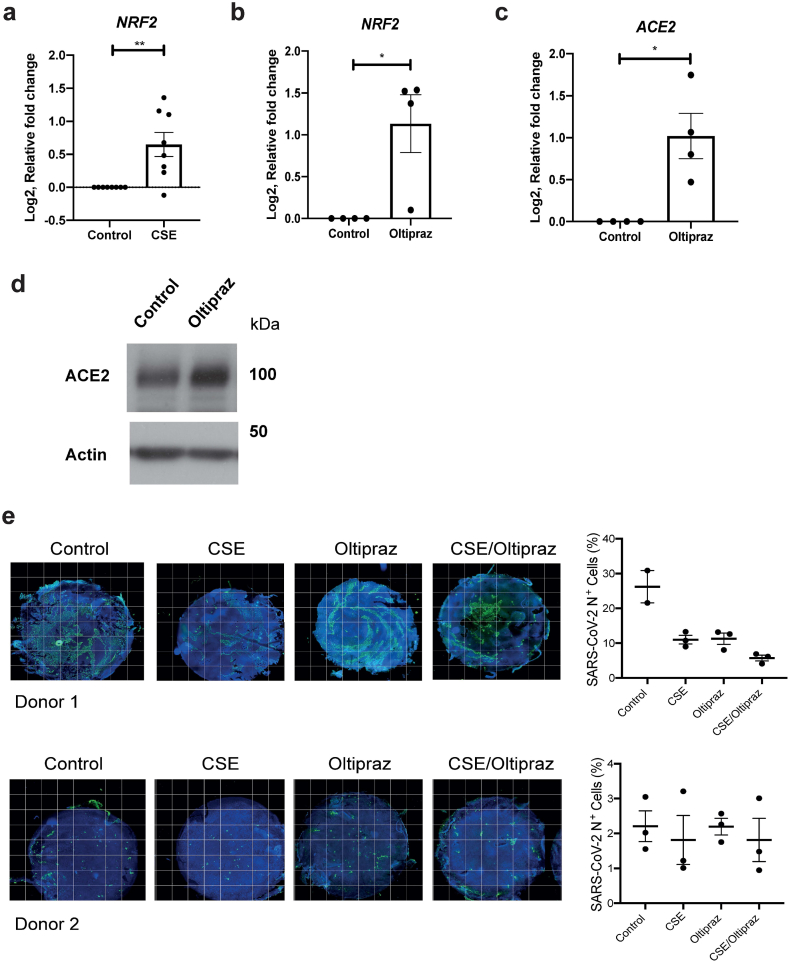
CSE and oltipraz increase ACE2 and NRF2 expression but not SARS-CoV-2 infection. **A.** CSE exposure induces NRF2 mRNA expression at 48 h. **B**. The NRF2 agonist Oltipraz increases NRF2 mRNA **C.** Oltipraz increases flACE2 mRNA expression. D. Oltipraz increases flACE2 protein expression (ACE2 antibody Ab15348). RT-PCR data shows log2 relative fold-change in expression from n = 7 independent experiments (Mann-Whitney, **P < 0.01, *p < 0.05). Representative Western blot from 3 independent experiments. **E.** Fluorescent image analysis of whole transwell ALI cultures show that for Donors 1 and 2, CSE or NRF2 agonists, alone or in combination, did not result in increased infection. Each datapoint represents a single well and infection was with 1 × 10^4^ TCID50 of B.1.1.7 SARS-CoV-2. Representative microscopy montages show the entire ALI transwell. Scale bar: 500 μm. Infection was quantitated and presented as percentage of cells infected for each condition. Control well for Donor 1 in this panel were shared with a previously published experiment [33].

Nuclear factor erythroid 2–related factor 2 (NRF2) is the master transcriptional regulator of the cellular antioxidant response and already a focus of therapeutic efforts to counteract epithelial oxidative stress in COPD. NRF2 agonists have also been proposed as therapeutics for COVID-19 [47]. In our experiments, CSE treatment of ALI cultures led to the expected increase in NRF2 as well as ACE2 upregulation (Fig. 5a). Further, oltipraz, a KEAP1 inhibitor and NRF2 agonist already in Phase III clinical trials, increased both ACE2 mRNA expression and flACE2 protein (Fig. 5b–d). This was a consistent finding in two donors – a non-smoker (Donor 1) and an individual with COPD (Donor 2), (Supplementary Fig. 8a). Despite elevating flACE2 levels (Supplementary Fig. 8a), oltipraz did not increase SARS-CoV-2 ((B1.1.7 variant) infection (Fig. 5e, Supplementary Fig. 8b). As noted elsewhere there is considerable inter-experimental variation in the efficiency SARS-CoV-2 infections in primary HBECs [9]. We therefore undertook multiple replicates from two donors and showed that there was no increase in infection with either CSE (consistent with Fig. 3C) or oltipraz (Fig. 5e, Supplementary Fig. 8b). We also assessed the impact of combined treatment with oltipraz and CSE. Again, despite induction of ACE2 (Supplementary Fig. 8a), there was no increase in SARS CoV-2 infectivity. Therefore, smoking and KEAP1 inhibition both increase flACE2 expression but do not lead to an increase in SARS-CoV-2 infection.

## 4 Discussion

There has been intense interest in the link between cigarette smoking and COVID-19. Smoking is a major cause of COPD, and current smokers or individuals with COPD are more at risk of severe COVID-19 infections and death [24,26]. Molecular epidemiological studies have linked COPD with increased expression of ACE2 [18,20,21]. Further, bulk and single cell RNA-Seq datasets comparing smokers and never-smokers have consistently shown that cigarette smoking (acute and chronic) leads to an increase in ACE2 expression, although these studies do not discriminate between ACE2 isoforms [17,19,48].

The elevated ACE2 expression in smokers was suggested to increase efficiency of infection [17,32]. However, the available epidemiological data suggests that smokers and non-smokers have similar risks of infection, but that those smokers or ex-smokers with cardiorespiratory end-organ damage (COPD, cardiovascular disease) are more likely to have severe infections or die from COVID-19 [24,27,29,30].

We have explored the link between smoking, ACE2 and SARS-CoV-2 infection *in vitro* using the optimal system to understand the earliest stage of *in vivo* infection – direct infection of differentiated primary human bronchial airway epithelial cells (HBECs) at the air-liquid interface (ALI). This system is ideal because the organotypic pseudostratified epithelium recapitulates the ‘*in vivo*’ microenvironment (air-liquid interface; polarised, ciliated apical surface) that is the first point of infection for the virus and, crucially, can be directly infected with virus at the apical surface like *in vivo*. For this purpose ALI cultures have advantages over differentiated alveolar organoids that need to be extracted from Matrigel for exposure to compounds or CSE. Alveolar organoids are not the first site of infection and need to be mechanically disrupted to perform luminal surface infection [49].

We demonstrate that cigarette smoke induces ACE2 expression in HBECs using multiple approaches – RT- PCR, western blotting and immunofluorescence. This finding is consistent with the molecular epidemiology data linking ACE2 expression and smoking. Given the difficulties reproducing ACE2 protein detection in clinical specimens [3,10], it is particularly important to have demonstrated upregulation of ACE2 protein at the cell surface – where it can act as a receptor for SARS-CoV-2. Importantly, in our experiments, despite the increased expression of ACE2, CSE does not alter cellular infection. This is also consistent with epidemiological data suggesting smoking is not a major risk factor for infection.

One possible explanation for these results was that CSE upregulates a truncated isoform of ACE2 – dACE2 - lacking the SARS-CoV-2 binding domain. However, we demonstrate that CSE predominantly induces flACE2, despite HBECs being competent to upregulate dACE2 in response to IFNα.

Our results differ from recent data that did not detect an increase in ACE2 mRNA/protein in response to cigarette smoke but nevertheless suggested that smoking increases viral infection [32]. The discrepancies are likely to reflect differences in the smoking exposure protocols used. We added CSE to the basal chamber of the transwell while cells are maintained at ALI. CSE is a well-established reagent used extensively in respiratory and cardiovascular research [50,51]. Further, it recapitulated the increase in ACE2 reported in clinical specimens and there was no evidence of toxicity. There are a number of optimised methods to directly expose ALI cultures to cigarette smoke that use customised apparatus. For example, Gindele et al. exposed differentiating ALI cultures to cigarette smoke over 28 days using the P.R.I.T. ExpoCube to nebulise smoke directly onto transwell inserts to accepted industry standards for toxicity studies (ISO 3308) [52]. This is a significant exposure to cigarette smoke and led to a clear induction of ACE2 (Supplementary Fig. 9). However, the ExpoCube or similar is not readily compatible with Class 3 facilities nor appropriate for experiments in which there would be a risk of nebulising SARS-CoV-2.

Purkayastha and others referred to Gindele et al.‘s work as a basis for their protocol but used a simple headspace protocol rather than the ExpoCube and performed exposure for only 3 min/day over 4 days. It may be that this modest exposure explains the lack of ACE2 induction [20,52]. The reasons for the discrepancy in terms of cellular infection are uncertain. We note that we performed our measures of infection based on imaging whole transwells and unbiased flow cytometry rather than selected high-powered fields. Further, our data reflects the epidemiology discussed above.

Our protocol was based on others modelling the impact of cigarette exposure through cigarette/CSE pretreatment and then viral infection [32,[53], [54], [55]]. However, we acknowledge that others have also combined CSE and virus [54], a variation that was impossible in a CL3 laboratory.

NRF2 agonists theoretically may act as antioxidants in COPD and have also been suggested as therapeutic agents in COVID-19 [47]. Oltipraz, an NRF2 agonist already in Phase 3 clinical trials, induced ACE2 expression in HBECs but, as with the CSE experiments, this was not associated with an increase in SARS-CoV-2 infection. This is consistent with the notion that ACE2 is not the key factor limiting cellular infection. Our data suggests that increasing flACE2 in ALI cultures has no impact on cellular infection and therefore that physiological levels of flACE2 expression may not be a key factor limiting SARS-CoV-2 infection. There are limited available data on this issue. Importantly, ACE2 expression is polarised at the apical/ciliated surface of differentiated pseudostratified respiratory epithelium, so the experiments using non-physiological undifferentiated cell lines transduced with ACE2 have limited *in vivo* relevance. Strategies to reduce ACE2 expression to protect against SARS-CoV-2 infection could be counterproductive if higher ACE2 in the distal airway is protective against acute lung injury [6,[56], [57], [58]]. Further, there is some evidence that TMPRSS2 expression may be more of limiting factor depending on the variant [59].

Nicotine was also being assessed as a potential protective agent in COVID-19 infection (ClinicalTrials.gov Identifier NCT04583410), based on early epidemiological observations suggesting smokers may be protected against SARS-CoV-2 infection as well as preclinical studies on submerged HBECs suggesting nicotine may increase ACE2 mRNA expression [41,60]. Our data show that nicotine does not significantly alter either ACE2 or CHRNA7 mRNA expression in differentiated HBECs after 48 h treatment.

Our studies have limitations. We deliberately focus on the conducting airways and the initial phases of infection rather than the later stage which are important for COVID-19 morbidity and mortality. Further, smoking has important systemic impacts that cannot be modelled in airway cultures. In that context it is possible that CSE in the basal media more closely mimics the sustained systemic impact of smoking as used in many years of research on endothelial pathology. In terms of assaying cellular infection, we have gone to great lengths to have unbiased quantitation of whole transwell infection rather than selected fields of view. We acknowledge that, while primary ALI cultures have profound advantages, it is possible that subtle changes in the efficiency of infection are not detected because of the well-to-well variation typical of experiments using primary cells. We have assayed cellular infection using protein expression rather than quantifying viral particles using RT-PCR or combining both approaches. Finally, a strength of this study is the use of SARS-CoV-2 rather than pseudovirus. The spike mutation profile has, of course, evolved considerably since the wild type and B1.1.7 variants used in this experimental work were prevalent. Variants differ in their binding to ACE2 binding [61] and the impact of this on in the context of smoke exposure is uncertain.

Overall, our data are entirely consistent with the documented epidemiology of SARS-CoV-2 infection. Individuals with smoking-related chronic respiratory or cardiovascular disease are more vulnerable to severe COVID-19. However, current smokers have a similar susceptibility to SARS-CoV-2 infection as the general population. We show that the airway epithelial response to cigarette smoke is associated with an increase in full length ACE2 – the key SARS-CoV-2 receptor - but not an increase in the efficiency of cellular infection. Therapeutic strategies that increase ACE2 receptor expression in the conducting airways are unlikely to increase cellular infection.

## Author contribution statement

Linsey Porter; Wenrui Guo; Thomas Crozier; Edward Greenwood: Conceived and designed the experiments; Performed the experiments; Analyzed and interpreted the data; Wrote the paper. Brian Ortmann; Daniel Kottmann: Contributed reagents, materials, analysis tools or data. James Nathan; Paul Lehner; Frank McCaughan: Conceived and designed the experiments; Analyzed and interpreted the data; Wrote the paper. Ravindra Mahadeva: Conceived and designed the experiments; Analyzed and interpreted the data; Contributed reagents, materials, analysis tools or data; Wrote the paper.

## Funding statement

Paul Lehner was supported by 10.13039/501100002927Addenbrooke's Charitable Trust, Cambridge University Hospitals [15/20 A]; 10.13039/100010269Wellcome Trust [084957/Z/08/Z]; 10.13039/501100000265Medical Research Council [MR/V011561/1].

Daniel Kottman was supported by a Medical Research Council studentship [MR/N013433/1].

Dr Frank McCaughan was supported by 10.13039/501100000849National Centre for the Replacement, Refinement and Reduction of Animals in Research [NC/S001204/1]; Dr Frank McCaughan and Dr Linsey Porter were supported by the 10.13039/100009855Roy Castle Lung Cancer Foundation [2015/10/McCaughan].

## Data availability statement

Data included in article/supplementary material/referenced in article.

## Declaration of competing interest

The authors declare no conflict of interest.

## Figures and Tables

**Table:** 

REAGENT or RESOURCE	SOURCE	IDENTIFIER
Antibodies		
Sheep SARS-CoV-2	MRC-PPU	DA114
AF488 donkey anti-sheep	Jackson ImmunoResearch	713-545-147
Goat anti-rabbit AF555	Invitrogen	A32732
Goat anti-mouse AF488	Invitrogen	A32723
Rabbit ACE2	Abcam	ab228349
Rabbit ACE2	Abcam	Ab15348
Rabbit ACE2	Abcam	ab108209
Mouse Alpha-tubulin	Santa Cruz	sc-32293
Mouse acetylated tubulin	Sigma	T7451
Mouse Muc5AC	Invitrogen	MA5-12178
Mouse SARS-CoV-2 (COVID-19) spike antibody [1A9]	Genetex	GTX632604
Mouse beta-actin	Santa Cruz	sc-69879
Bacterial and virus strains		
SARS-CoV-2		SARS-CoV-2/human/Liverpool/REMRQ0001/2020
SARS-CoV-2 B.1.1.7		SARS-CoV-2 England/ATACCC174/2020
		
		
		
Biological samples		
Normal primary human bronchial epithelial cells	Lonza	CC-2540
Primary human bronchial epithelial cells from a COPD donor derived from proximal brushings	Cambridge University Hospitals NHS Trust	
		
		
		
Chemicals, peptides, and recombinant proteins		
		
		
		
		
		
Critical commercial assays		
AnnexinAPC/PI apoptosis	**88**–**8007**–**72**	eBioscience
		
		
		
		
Deposited data		
		
		
		
		
		
Experimental models: Cell lines		
A549	ATCC	CCL-185
Calu3	ATCC	HTB-55
HEK293T	ATCC	CRL-3216
		
		
Experimental models: Organisms/strains		
		
		
		
		
		
		
Oligonucleotides		
ACE2_F CGAAGCCGAAGACACTGTTCTA		
ACE2_R GGGCAAGTGTGGACTGTTCC		
dACE2_F GGAAGCAGGCTGGGACAAA		
dACE2_R AGCTGTCAGGAAGTCGTCCATT		
TBP_F AGTGAAGAACAGTCCAGACTG		
TBP_R CCAGGAAATAACTCTGGCTCAT		
TMPRSS2_F CTGCTGGATTTCCGGGTG		
TMPRSS2_R TTCTGAGGTCTTCCCTTTCTCCT		
FOXJ1_F TGGATCACGGACAACTTCTGCTA		
FOXJ1_R CACTTGTTCCAGAGACAGGTTGTGG		
MUC5B_F CCTGAAGTCTTCCCCAGCAG		
MUC5B_R GCATAGAATTGGCAGCCAGC		
Recombinant DNA		
		
		
		
		
		
Software and algorithms		
GraphPad Prism 8	GraphPad Software	
Fiji	Image J	
Adobe Photoshop	Adobe	
		
		
Other		

## Appendix A Supplementary data

Multimedia component 1 **Suppl**Fig. 1**CSE upregulates *ACE2* expression relative to control wells**. **A.** Dose response of ACE2 to 10 and 20% CSE exposure. There was an increase of ACE2 at both doses, so the lower concentration was used for ongoing experiments. RT-PCR data shows log2 relative fold-change in expression from n = 5 independent experiments, (Mann-Whitney, **p < 0.01). **B**. Time-course of ACE2 and TMPRSS2 induction in response to 10% CSE. ACE2 was significantly increased at 48h. There was no significant change in TMPRSS2 expression. RT-PCR data shows log2 relative fold-change in expression from n = 3 (24h) or n = 5 (48h) independent experiments, (Mann-Whitney, **p < 0.01). **Suppl**Fig. 2**CSE upregulates *ACE2* expression relative to control wells**.**A.** Differentiated HBECs (Donor 1) cultured at the ALI for 28 days were stained with antibodies against ACE2 (red, 21115-AP) and acetylated tubulin (green). **B.** Red fluorescence in the untreated well was reduced until ACE2 signal could not be detected and this threshold was then applied to CSE exposed wells. CSE exposed wells showed a persistent apical ACE2 signal. Data is representative of two independent experiments.**Suppl**Fig. 3**. Exposure to CSE induces ACE2 expression in differentiated HBECs derived from an individual with COPD (Donor 2). A**. CSE exposure (10%) for 48 h increases *ACE2* expression in differentiated COPD-derived HBECs relative to untreated controls. RT-PCR data shows log2 relative fold-change in expression from n = 4 independent experiments, (Mann-Whitney, *p < 0.05). **B.** CSE exposure (10%) for 48 h also increases ACE2 protein expression relative to untreated control (Antibody: Ab15348). Calu-3 and A549-ACE2 are presented as the positive controls. Representative western blot from 4 independent experiments. **Suppl**Fig. 4**. Representative flow cytometric plots and histograms from CSE exposed and untreated HBEC cultures (Donor 1).** CSE exposure does not induce apoptosis in differentiated HBECs at ALI relative to control samples. HBECs at ALI exposed to CSE (10%) for 48 h were harvested and dual stained with AnnexinV and propidium iodide for flow cytometric analysis. Apoptotic cells are identified as being both AnnexinV and propidium iodide positive, incorporating both early and late stages of cell death. Data represents plots derived from one of two independent experiments. **Suppl**Fig. 5**. Nicotine treatment results in a modest and variable upregulation in ACE2 relative to CSE treatment (Donor 1)**. Nicotine treatment (1 μM) for 48 h also modestly increases ACE2 protein expression relative to untreated control. Calu-3 are presented as the positive control. Representative western blot from 3 independent experiments. WB performed using ACE2 N (Ab228349) or C-terminal (Ab15348) antibodies. **Suppl**Fig. 6**. CSE exposure does not significantly alter interferon-associated gene expression (Donor 1).** RT-PCR data shows log2 relative fold-change in expression from n = 7 independent experiments from 7 experiments, (Mann-Whitney, ns). **Suppl**Fig. 7**. CSE exposure does not promote an increase in dACE2 protein. Western blot showing 5 independent experiments showing the impact of CSE on flACE2/dACE2 expression. ACE2 antibody used ab15348. Suppl**Fig. 8**. Impact of CSE and oltipraz on flACE2 expression and cellular infection. A. Representative western blots from two donors. Calu-3a and b represent Calu-3 cells from different experiments. ACE2 antibody (Ab15348). B. Quantitation of immunofluorescence signal using microscopy as described in**Fig. 5E **- replicate experiment using cells at ALI on transwells from Donor 1 infected with 1x10**^**4**^**TCID50 of B.1.1.7 lineage SARS-CoV-2. Antibody used was SARS-CoV-2 nucleocapsid MRC PPU DA114.Suppl**Fig. 9**. Figure based on RNA-Seq data from Gindele et al, 2020 extracted from Gene Expression Omnibus (GSE135188). Small airway epithelial cultures (SAEC) at ALI were exposed to cigarette smoke (CS) or air three times a week during the differentiation phase, that is from the period from Day 0 to Day 28. ACE2 expression is significantly increased by CS exposure in 2/6 donors whilst shows a positive trend for increasing in 4/6 donors.** Multimedia component 1
