# Supplementary material for: Cigarette smoke preferentially induces full length ACE2 expression in differentiated primary human airway cultures but does not alter the efficiency of cellular SARS-CoV-2 infection
Source: Heliyon. 2023 Mar 11;9(3):e14383. doi: 10.1016/j.heliyon.2023.e14383 (PMC10005841; doi:10.1016/j.heliyon.2023.e14383)

Suppl Figure 1

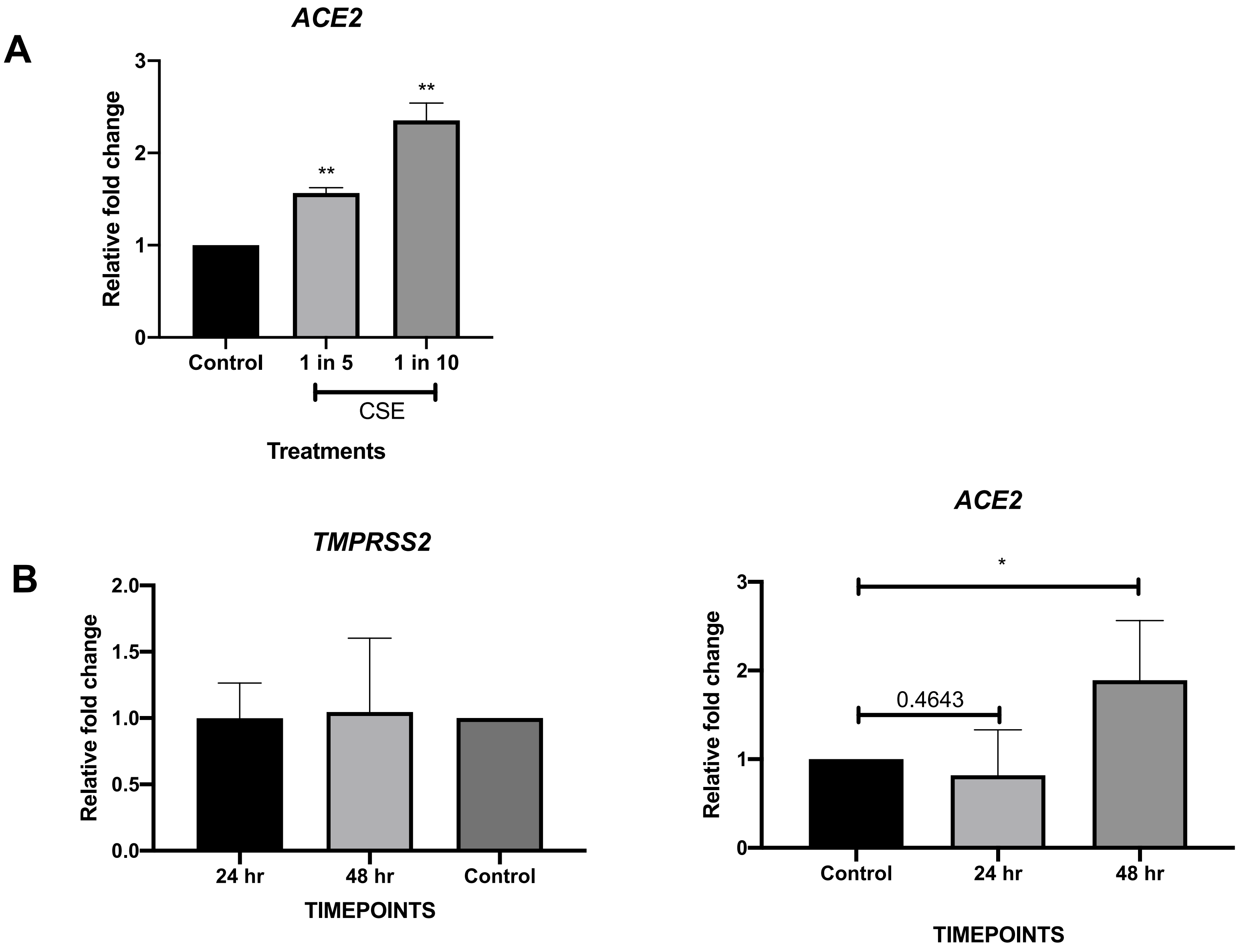

Suppl Figure 2

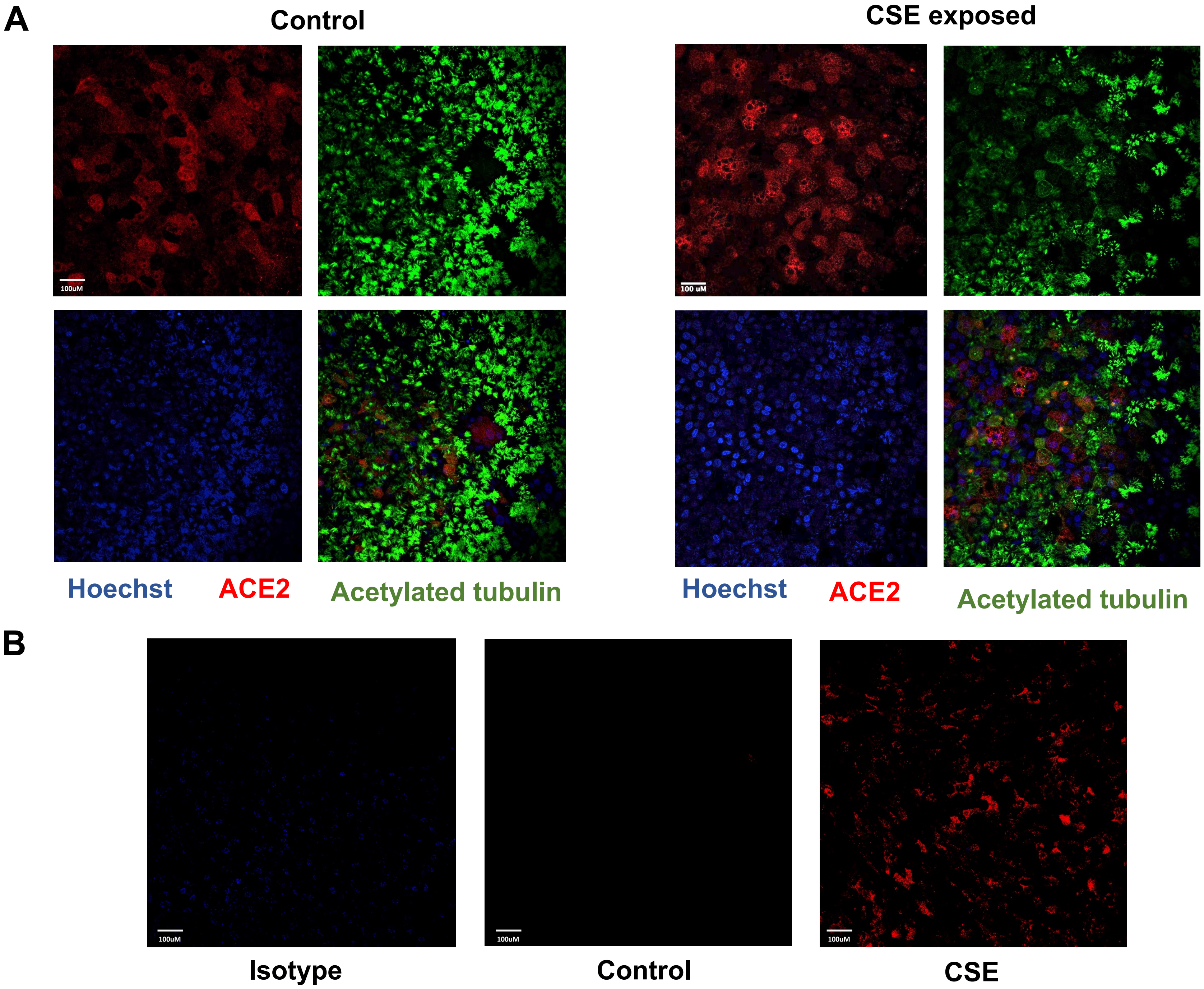

Suppl Figure 3

A

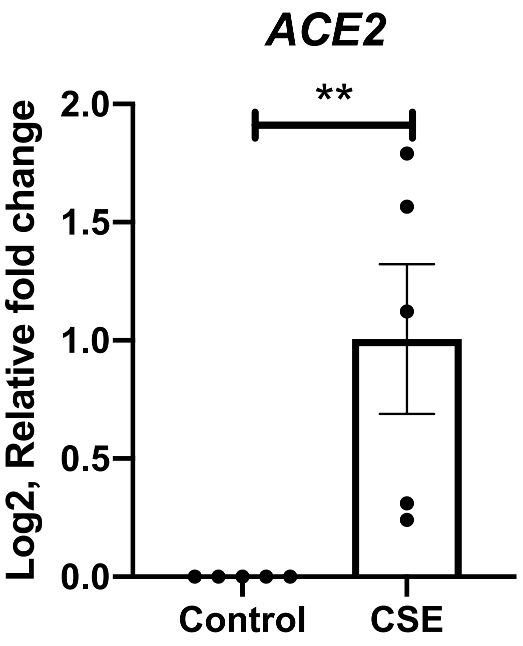

B

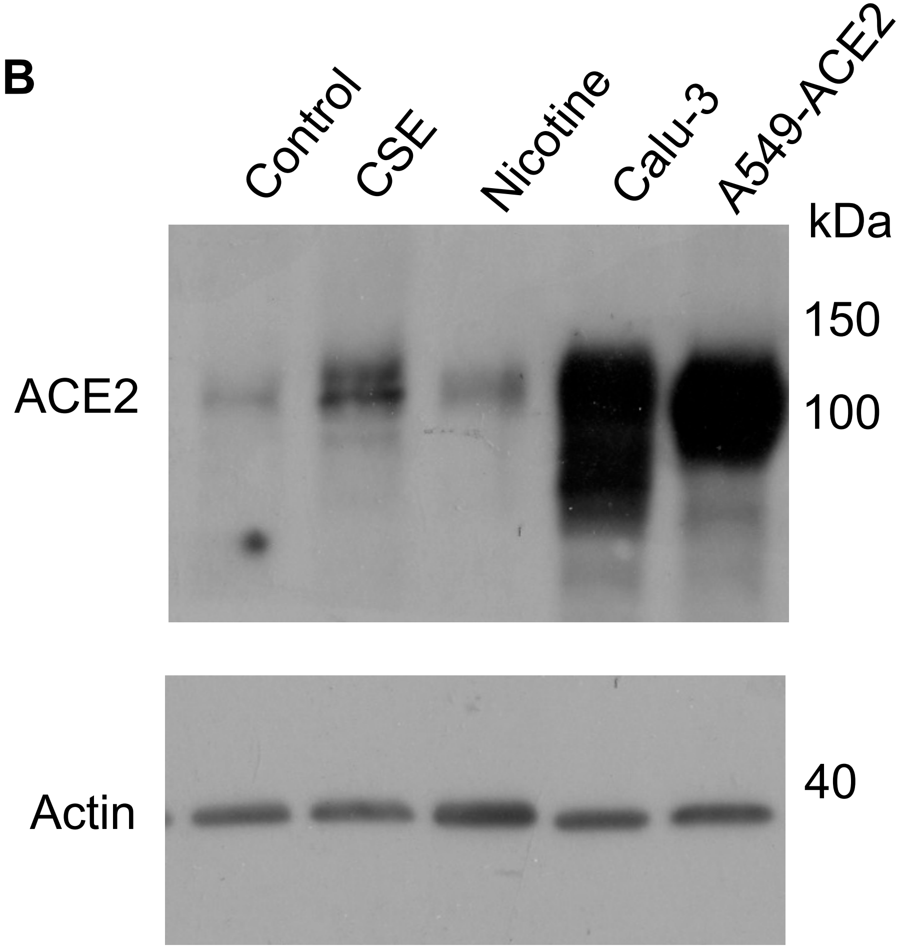

Suppl figure 4

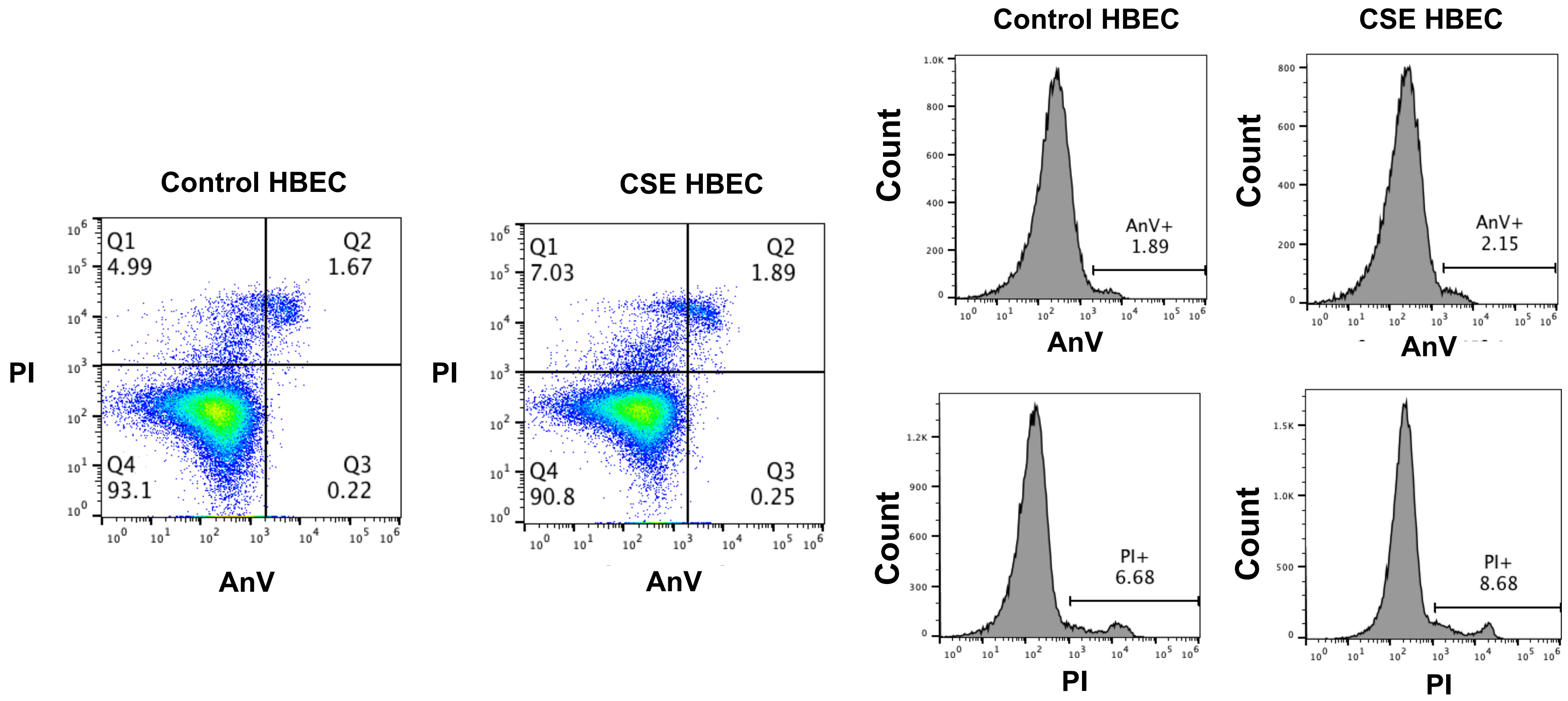

Suppl figure 5

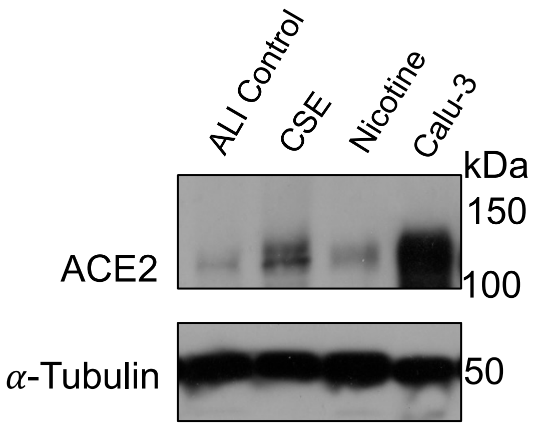

C-terminal ACE2 antibody

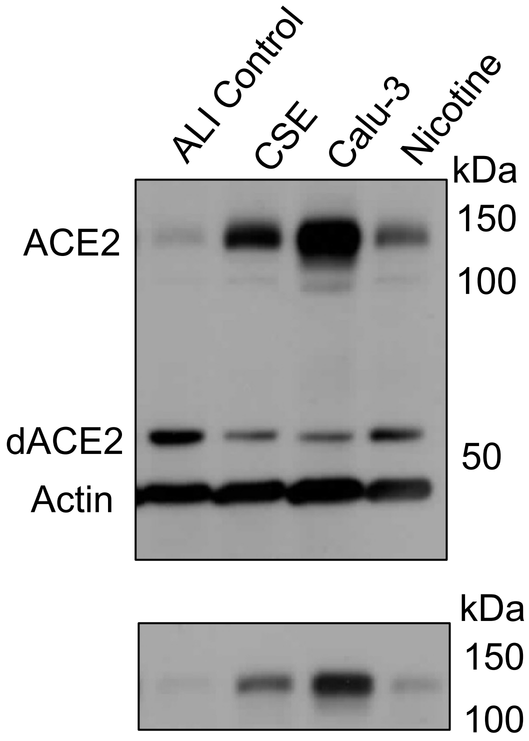

C-terminal ACE2 antibody

N-terminal ACE2 antibody

Suppl figure 6

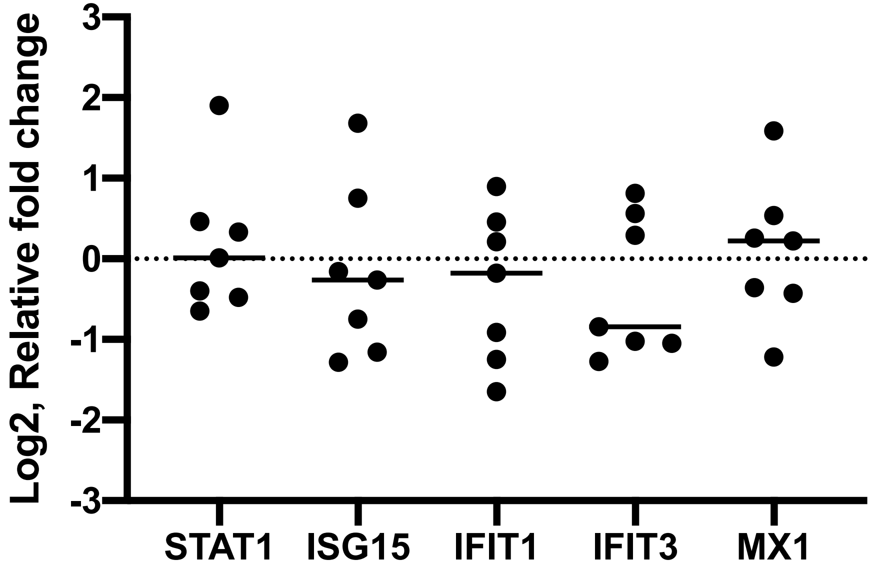

Suppl figure 7

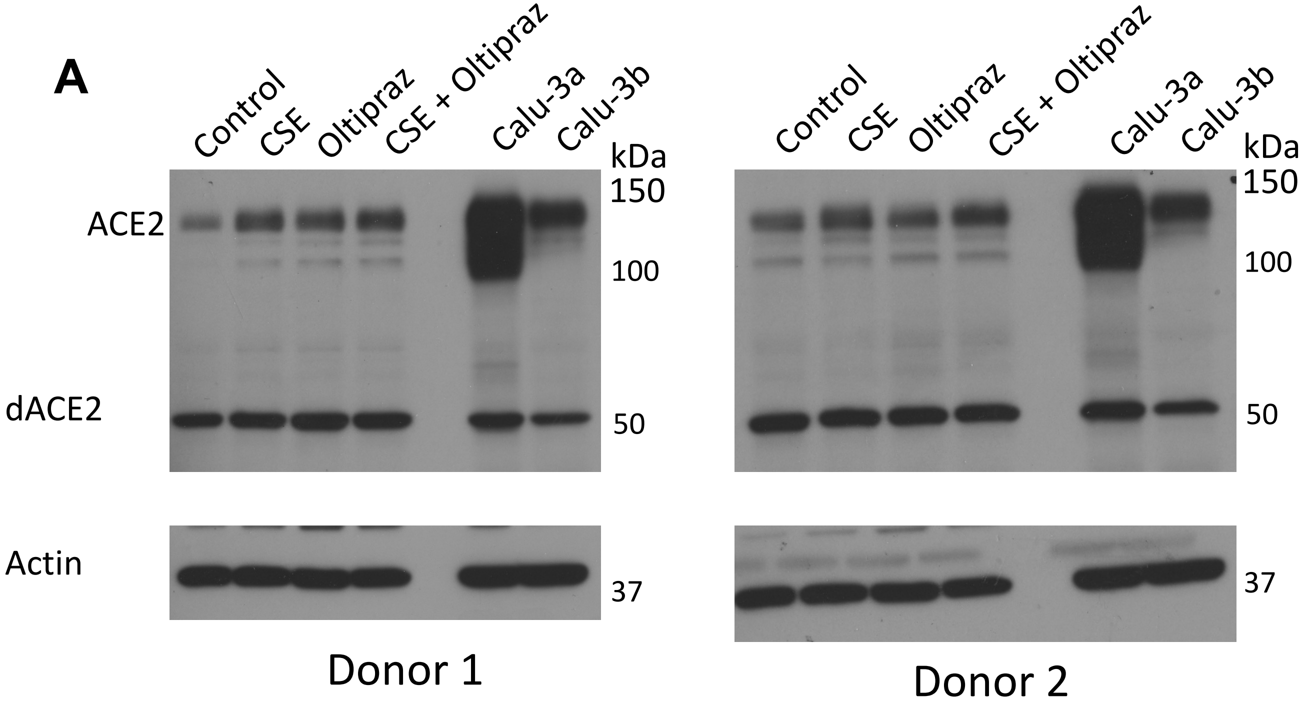

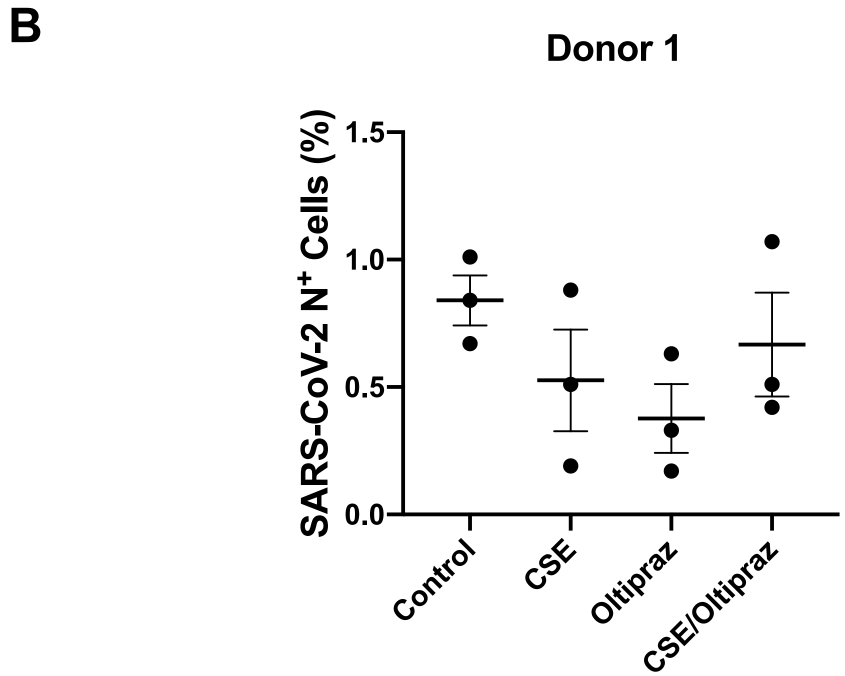

Suppl figure 8

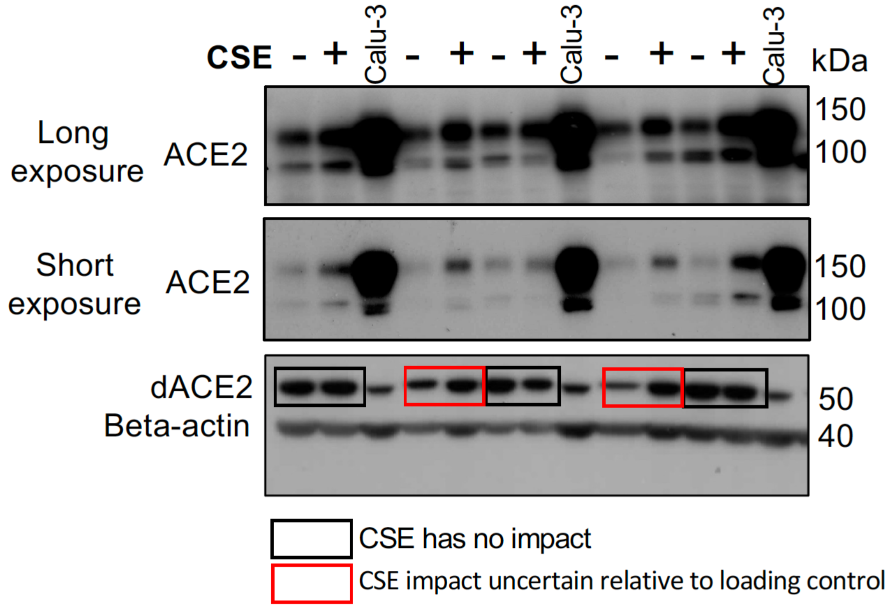

Suppl figure 8

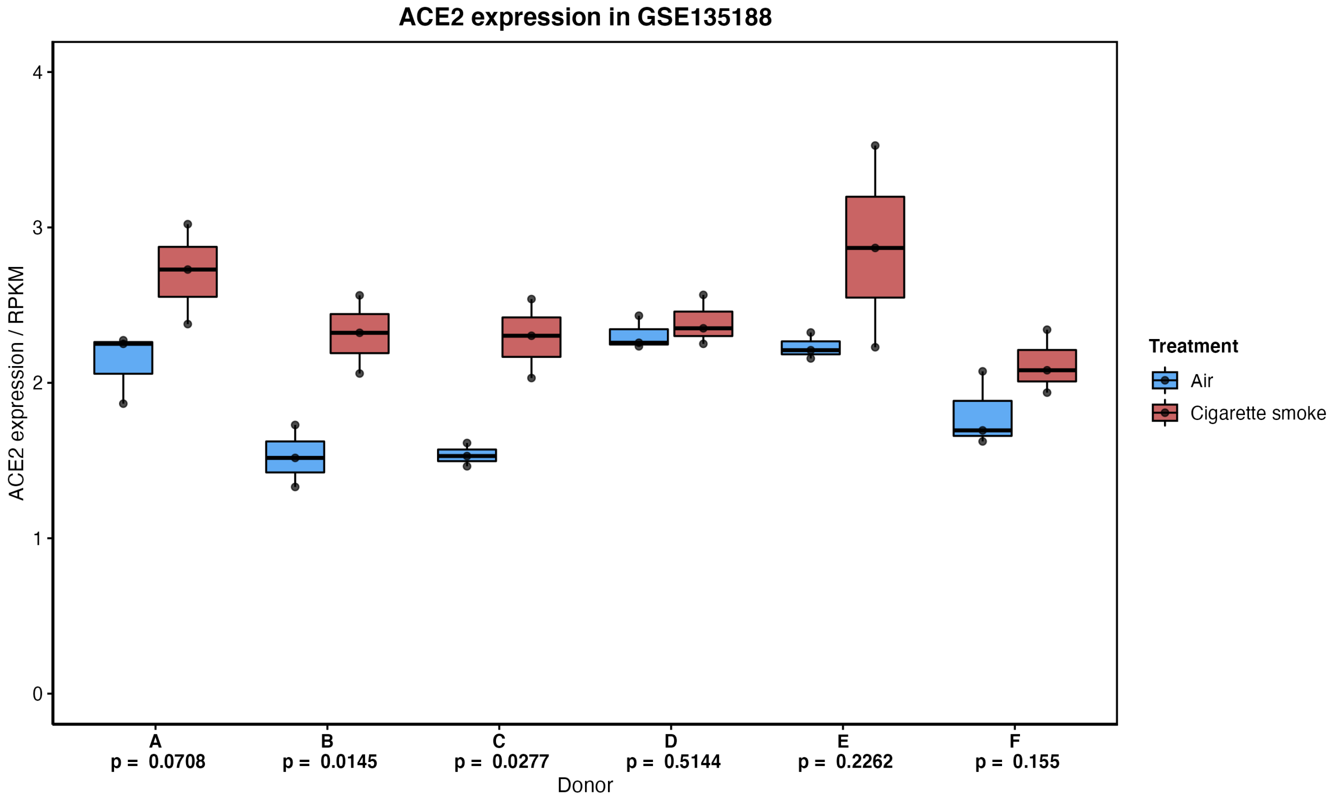

Supplement: Multimedia component 1 — SupplFig. 1CSE upregulates ACE2 expression relative to control wells. A. Dose response of ACE2 to 10 and 20% CSE exposure. There was an increase of ACE2 at both doses, so the lower concentration was used for ongoing experiments. RT-PCR data shows log2 relative fold-change in expression from n = 5 independent experiments, (Mann-Whitney, **p < 0.01). B. Time-course of ACE2 and TMPRSS2 induction in response to 10% CSE. ACE2 was significantly increased at 48h. There was no significant change in TMPRSS2 expression. RT-PCR data shows log2 relative fold-change in expression from n = 3 (24h) or n = 5 (48h) independent experiments, (Mann-Whitney, **p < 0.01). SupplFig. 2CSE upregulates ACE2 expression relative to control wells.A. Differentiated HBECs (Donor 1) cultured at the ALI for 28 days were stained with antibodies against ACE2 (red, 21115-AP) and acetylated tubulin (green). B. Red fluorescence in the untreated well was reduced until ACE2 signal could not be detected and this threshold was then applied to CSE exposed wells. CSE exposed wells showed a persistent apical ACE2 signal. Data is representative of two independent experiments.SupplFig. 3. Exposure to CSE induces ACE2 expression in differentiated HBECs derived from an individual with COPD (Donor 2). A. CSE exposure (10%) for 48 h increases ACE2 expression in differentiated COPD-derived HBECs relative to untreated controls. RT-PCR data shows log2 relative fold-change in expression from n = 4 independent experiments, (Mann-Whitney, *p < 0.05). B. CSE exposure (10%) for 48 h also increases ACE2 protein expression relative to untreated control (Antibody: Ab15348). Calu-3 and A549-ACE2 are presented as the positive controls. Representative western blot from 4 independent experiments. SupplFig. 4. Representative flow cytometric plots and histograms from CSE exposed and untreated HBEC cultures (Donor 1). CSE exposure does not induce apoptosis in differentiated HBECs at ALI relative to control samples. HBECs at ALI expos [file mmc1.pdf]
