# Supplementary material for: Cigarette smoke preferentially induces full length ACE2 expression in differentiated primary human airway cultures but does not alter the efficiency of cellular SARS-CoV-2 infection
Source: Heliyon. 2023 Mar 11;9(3):e14383. doi: 10.1016/j.heliyon.2023.e14383 (PMC10005841; doi:10.1016/j.heliyon.2023.e14383)
Supplement: Multimedia component 2 [file mmc2.docx]

**Supplementary Material**

**Air-liquid interface culture**

**Cell Lines**

Cell lines including A549 (ATCC; Cat# CCL-185, male), Calu3 (ATCC; Cat# HTB-55, male) HEK293T (ATCC; Cat# CRL-3216, female) (A549-ACE2, HEK293T-ACE2) have been used as negative or positive controls. HEK293T lines were maintained in RPMI supplemented with 10% FBS, 2 mM L-glutamine, pH 7.5, and 1 mM sodium pyruvate at 37°C in a 5% CO2.  A549 and Calu-3 cell lines were cultured in Dulbecco’s Modified Eagle’s Medium (DMEM) and Eagle’s Minimal Essential Medium (EMEM) respectively, supplemented as specified above.

QPCR

RNA was extracted using RNeasy Mini Kit (Qiagen) according to manufacturer’s instructions and quantified using a NanoDrop Spectrophotometer (ThermoFisher). cDNA synthesis was performed using a High-Capacity cDNA Reverse Transcription Kit (ThermoFisher). qRT-PCR was performed using Fast SYBR® Green Mix (ThermoFisher) alongside the following primers used for detecting expression of genes of interest: ACE2 Forward (5’-3’): CGAAGCCGAAGACCTGTTCTA, Reverse (5’-3’): GGGCAAGTGTGGACTGTTCC; dACE2 Forward (5’-3’): GGAAGCAGGCTGGGACAAA, Reverse(5’-3’): AGCTGTCAGGAAGTCGTCCATT; TBP (House keeper) Forward (5’-3’): AGTGAAGAACAGTCCAGACTG, Reverse (5’-3’): CCAGGAAATAACTCTGGCTCAT; TMPRSS2 Forward (5’-3’): CTGCTGGATTTCCGGGTG, Reverse (5’-3’) TTCTGAGGTCTTCCCTTTCTCCT; FOXJ1 Forward (5’-3’): TGGATCACGGACAACTTCTGCTA, Reverse (5’-3’) CACTTGTTCCAGAGACAGGTTGTGG; MUC5B Forward (5’-3’): CCTGAAGTCTTCCCCAGCAG, Reverse (5’-3’) GCATAGAATTGGCAGCCAGC. Samples were run in technical triplicates on a StepOne machine and relative differences in expression were determined using the comparative ΔC_T_ method and TBP used as the endogenous house-keeping control.

**SARS-CoV-2 infection**

The clinical isolate of SARS-CoV-2 viruses used in this study were SARS-CoV-2/human/Liverpool/REMRQ0001/2020(Lineage B.29) and SARS-CoV-2 England/ATACCC 174/2020 (Lineage B.1.1.7) [34, 35]. Stocks were sequenced before use and the consensus matched the expected sequence exactly. Viral titre was determined by 50% tissue culture infectious dose (TCID50) in Huh7-ACE2 cells.

For infection, the indicated dose of virus was diluted in PBS to a final volume of 50 µL and added to the apical chamber of the transwell of differentiated HBEC-ALI cultures for 2-3 hours prior to removal. At 72 hours post-infection HBEC-ALI apical surfaces were washed once with PBS, dissociated with TrypLE, and fixed in 4% formaldehyde for 15 minutes. Fixed cells were washed and incubated for 15 minutes at room temperature in Perm/Wash buffer (BD #554723). Permeabilised cells were pelleted, stained for 15 minutes at room temperature in 100 µL of sheep anti-SARS-CoV-2 nucleocapsid antibody (MRC-PPU, DA114) at a concentration of 0.7 µg/mL, washed and incubated in 100 µL AF488 donkey anti-sheep (Jackson ImmunoResearch #713-545-147) at a concentration of 2 µg/mL for 15 minutes at room temperature. Stained cells were pelleted and fluorescence staining analysed on a BD Fortessa flow cytometer.

**Immunofluorescence**

The ALI cultures were washed three times with PBS and fixed using 4% paraformaldehyde (PFA) for 15 minutes at room temperature before permeabilization with 0.3% Triton-X for 15 minutes. Cells were blocked for 1 hour in 5% Normal goat serum/1% Bovine serum albumin (BSA) at room temperature. Primary antibodies; anti-ACE2 antibody was initially Abcam 228349 but was discontinued part-way through this study and was then replaced with 21115-1-AP (Proteintech); Acetylated tubulin (T7451; Sigma); Muc5AC (MA5-12178; Invitrogen), SARS-CoV / SARS-CoV-2 (COVID-19) spike antibody (1A9) (GTX632604; Genetex), SARS-CoV-2 (COVID-19) Nucleocapsid antibody DA114 (MRC PPU) were added and incubated at 4 degrees overnight. Following several washes with Phosphate Buffered Saline and Tween-20 (PBS-T), cultures were incubated with secondary antibodies for 1 hour in the dark at room temperature before being washed a further three times before Hoechst staining (100 μg/ml) and mounting. Confocal images were captured using a Nikon C2 Confocal Microscope, magnification ×40 oil. Composite images were generated and analysed using Fiji. Immunofluorescent images were also captured using a Cellomics Arrayscan (ThermoFisher Scientific VTI) using 64 fields of view/transwell at x10 magnification and analysed using HCS. Studio 2.0 Client Software. Results are expressed as percent of infected cells according to AF488 positive staining. For Figure 5E (Donor 1 only) the controls were shared with a published experiment [33].
